# Myocardial Infarction With Non-obstructive Coronary Arteries: Risk Factors and Associated Comorbidities

**DOI:** 10.3389/fcvm.2022.895053

**Published:** 2022-05-02

**Authors:** Andrea Carlo Merlo, Alessandro Troccolo, Elisa Piredda, Italo Porto, Vered Gil Ad

**Affiliations:** ^1^Division of Cardiovascular Diseases, Department of Internal Medicine and Medical Specialties, University of Genoa, Genoa, Italy; ^2^Cardiology Unit, DICATOV - Cardiothoracic and Vascular Department, Istituto di Ricovero e Cura a Carattere Scientifico (IRCCS) Ospedale Policlinico San Martino, Genoa, Italy

**Keywords:** myocardial infarction, MINOCA, coronary artery disease, risk factor, comorbidities

## Abstract

Myocardial infarction with non-obstructive coronary arteries (MINOCA), despite a lower burden of coronary atherosclerosis, has a non-negligible prognostic impact. The label of MINOCA includes an array of different aetiologies and pathologic conditions, thus the identification of the underlying disease is crucial to patient management. Myocardial infarction with obstructive coronary artery disease and MINOCA share only some risk factors and comorbid conditions. While traditional cardiovascular risk factors have a lower prevalence in MINOCA patients, atypical ones—e.g., anxiety, depression, and autoimmune diseases—are much more frequent in this population. Other conditions—e.g., pregnancy, cancer, and anti-cancer therapy—can predispose to or even induce MINOCA through various mechanisms. The evidence of such risk factors for MINOCA is still scarce and contradicting, as no randomised controlled trials exist in this field. In our work, we performed a review of registries, clinical studies, and case reports of MINOCA, in order to summarise the available data and analyse its possibile pathogenic mechanisms.

## Introduction

Myocardial infarction with non-obstructive coronary arteries (MINOCA) is defined as myocardial infarction (MI)—according to the universal definition criteria—in the absence of obstructive coronary arteries on angiography—i.e., no coronary artery stenosis ≥50% in any potential infarct-related artery—, provided that no overt specific cause for the presentation has been found (1, 2). Non-obstructive coronary arteries include normal or near-normal coronary arteries—i.e., with no ≥30% stenosis—and mild coronary atherosclerosis—i.e., stenosis >30% but <50%. The 50% cutoff is partly controversial, owing to inter- and intra-observer variability in stenosis estimation on traditional coronary angiography as well as possible changes in vessel diameter resulting from a fluctuating vasomotor tone and an unstable plaque (3). Notably, MINOCA accounts for about 6–9% of patients with MI (4, 5). However, since the label of MINOCA includes an array of different etiologies and underlying pathologic conditions, it should be considered a *working diagnosis*, which highlights the importance of identifying its specific mechanisms in order to inform patient management.

There are currently few data on predisposing factors and comorbidities associated with MINOCA. In this work, we performed an extensive search of the literature on PubMed and Google Scholar using the algorithm “MINOCA” AND (“typical” OR “atypical” OR “unusual”) AND (“risk factors” OR “comorbidities”). We considered studies, case reports/series, registries, and meta-analyses in English language providing data about MINOCA, particularly its association with typical and unusual risk factors as well as comorbid conditions. Finally, among the retrieved results, we identified and selected the most recent articles—including 11 observational studies, three case reports/series, one registry, and one meta-analysis—and reported their most significant findings.

## Classification

**Figure 1:**
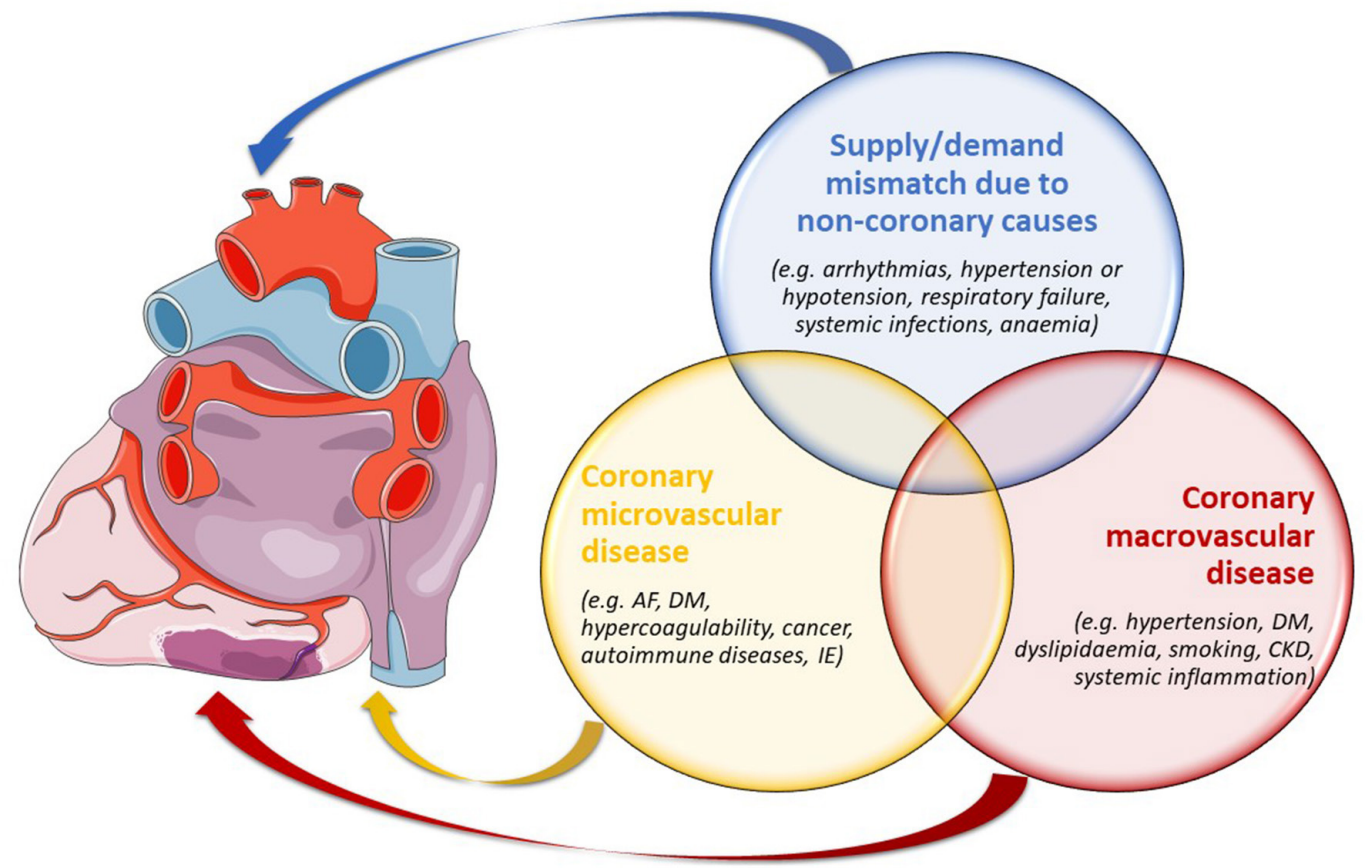
The principal types of MINOCA as per aetiologies and pathogeneses, potentially overlapping between one another. AF, atrial fibrillation; CKD, chronic kidney disease; DM, diabetes mellitus; IE, infective endocarditis.

Based on the underlying mechanism, MINOCA may be classified into two main subgroups, i.e., epicardial and microvascular (5). Furthermore, other forms of type 2 MI should be considered in the differential diagnosis. Despite myocardial ischaemia likely playing a role in its pathogenesis, the inclusion of takotsubo syndrome (TTS) in MINOCA is still debated, as it shows unique features (1). A summary of MINOCA classification is reported in Figure 1.

### Epicardial Causes

*Coronary plaque disease* may explain almost half of cases of MINOCA, as documented by intracoronary imaging (6). Despite the absence of critical coronary stenoses, the two main pathologic findings are plaque rupture and plaque erosion, just like in type 1 MI. MINOCA may depend on thrombosis, thromboembolism, superimposed vasospasm, or a combination of these processes, and spontaneous thrombolysis has been suggested to explain patency of the coronary arteries (5). Thrombosis complicating a calcified nodule may constitute another possible cause of MINOCA on intracoronary imaging (7).

*Coronary artery spasm* reflects a vascular smooth muscle hyper-reactivity to specific triggers, which may be endogenous or exogenous (8). Provocative tests with intracoronary ergonovine or acetylcholine were reported positive—i.e., ST-segment changes and angina with ≥90% coronary artery diameter reduction—in up to 46% of patients presenting with MINOCA and suspected coronary vasomotor abnormalities, also defining a subpopulation with worse outcomes (9, 10).

*Spontaneous coronary artery dissection* (SCAD) may present as either a proper dissection or an intramural haematoma without intimal tear (11). SCAD is responsible for about 1–4% of acute coronary syndromes (ACSs) overall and 25–35% of MIs in women ≤ 50 years of age (12, 13). It predominantly affects young white females, most often occurring in patients with a low prevalence of traditional cardiovascular risk factors (14). The left anterior descending artery is more frequently involved, probably owing to haemodynamic peculiarities (15).

### Microvascular Causes

*Coronary microvascular spasm*, indicated by ST-segment changes and angina with no or <90% coronary artery diameter reduction, is reported in about 25% of patients with MINOCA. Acetylcholine provocation testing can portend the diagnosis (10). Whereas long-term mortality rate seems very low, angina often persists despite medical therapy, having a negative impact on the quality of life (16).

*Coronary thromboembolism* usually involves the microcirculation, although large thrombi or emboli may abruptly interrupt coronary flow at epicardial level. Coronary thromboembolism may result from coagulation disorders—i.e., thrombophilia—as well as various embolic sources and predisposing conditions, including atrial fibrillation, mechanical valve prostheses, and right-to-left interatrial shunt. Moreover, MINOCA may rarely result from septic coronary embolism in patients with infective endocarditis (5).

### Takotsubo Syndrome

Takotsubo syndrome (TTS) often presents as an acute, reversible heart failure in postmenopausal women, in the absence of obstructive coronary artery disease (CAD) (17). Since TTS may mimic both myocarditis and type 1 MI, the differential diagnosis is extremely challenging and is usually ascertained after transthoracic echocardiography, coronary angiography, and ventriculography. Contrast-enhanced cardiac magnetic resonance shows a typical pattern and may be useful in unclear cases (18). Pathophysiology of TTS is multifaceted and may significantly vary between patients. Among possible mechanisms, multi-vessel epicardial spasm, catecholamine-induced myocardial stunning, spontaneous lysis of coronary thrombus, and acute microvascular spasm appear the most plausible. Particularly, diffuse coronary microvascular dysfunction is a common determinant of TTS (19). Long-term prognosis is generally good, although in-hospital mortality reaches 8%, mainly due to complications such as heart failure, ventricular arrhythmias, and left ventricular free wall rupture (20).

## Epidemiology and Prognosis

Owing to a significantly distinct pathophysiology, MI with concomitant obstructive CAD and MINOCA share only some risk factors and comorbid conditions. Moreover, each MINOCA subtype does have its own peculiarities.

Compared with patients with obstructive CAD, those with MINOCA are younger, less often smokers and diabetic, and have lower values of total and low-density lipoprotein cholesterol. They more frequently present with non-ST-segment elevation MI, a greater left ventricular ejection fraction, and lower Killip classes, showing reduced rates of in-hospital death. Notably, MINOCA is significantly associated with pulmonary disease, which marks its different epidemiological profile (21). Despite a lower burden of coronary atherosclerosis, MINOCA has a non-negligible prognosis since, according to emerging data, long-term mortality and rates of major adverse cardiovascular events (MACE) are as high as in MI with obstructive CAD (9, 22). Nonetheless, these results have been refuted by other studies and warrant further corroboration (4, 23). Contradictory findings may result from the extreme variability of the populations enrolled in the studies. However, it seems evident that the prognosis of MINOCA mainly depends on the underlying mechanism, also considering high-risk subsets of patients—e.g., those with coronary plaque rupture or epicardial vasospasm (24).

## Risk Factors and Comorbidities

**Figure 2:**
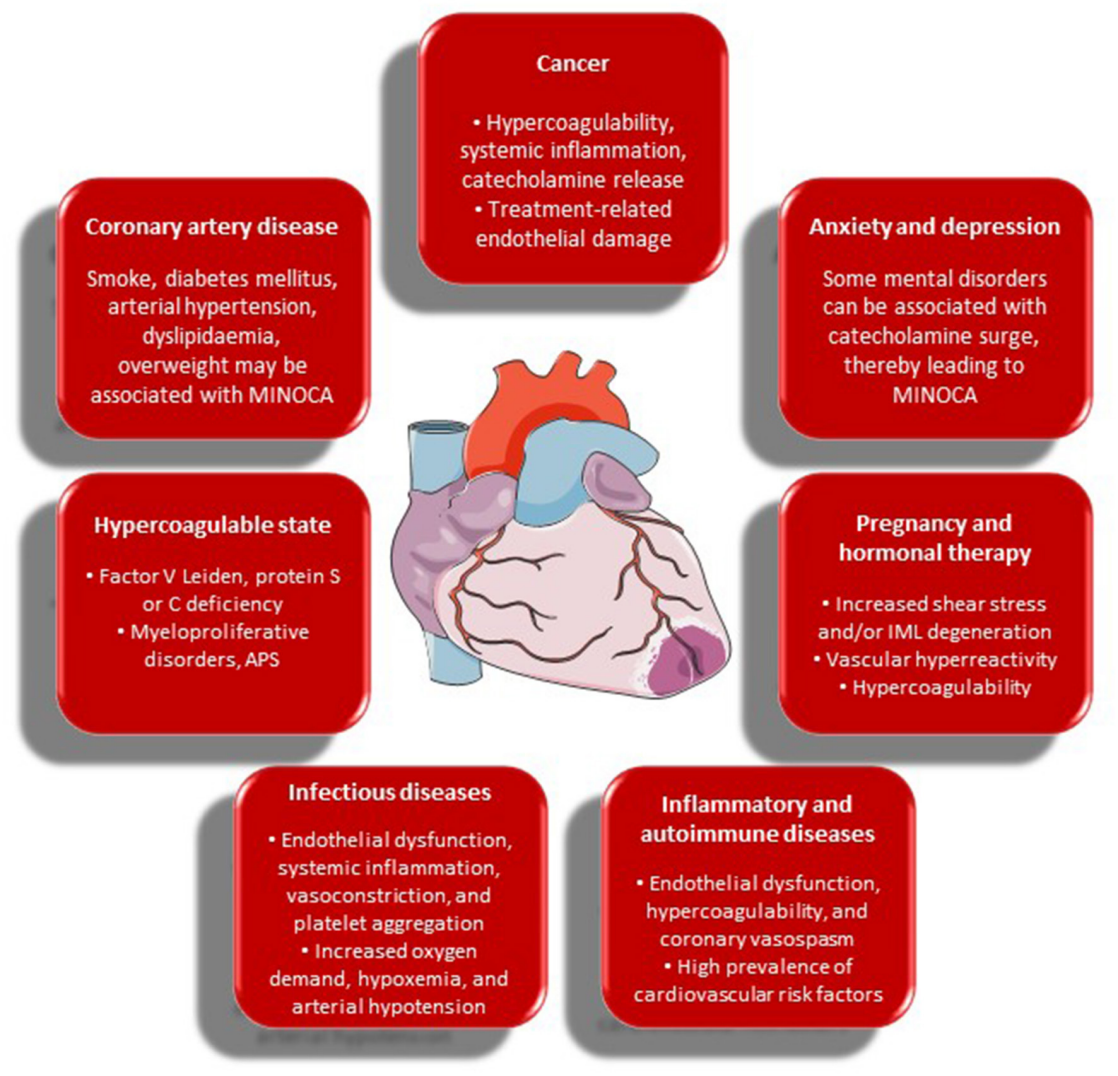
Risk factors and comorbidities of MINOCA and their possible contributory role in its occurrence. APS, antiphospholipid syndrome; IML, intima-media layer; MINOCA, myocardial infarction with non-obstructive coronary arteries.

The main links between MINOCA and its risk factors and comorbidities, as discussed in detail below, are presented in Figure 2.

- *Cardiovascular risk factors*: compared to MI with obstructive CAD, the prevalence of conventional cardiovascular risk factors is significantly lower in MINOCA patients (25). According to the VIRGO study, smoking, arterial hypertension, diabetes mellitus, dyslipidaemia, obesity, family history of CAD, and prior MI or peripheral artery disease showed a decreased frequency within the MINOCA population. Conversely, unconventional risk factors—e.g., history of illicit drug use, known renal dysfunction, autoimmune diseases, and venous thromboembolism—did not differ between the groups (22).
- *Hypercoagulable state*: coronary thrombosis may arise from hereditary or acquired thrombophilia, while coronary emboli may occur from coronary or systemic arterial thrombi (5). Paradoxical embolism can be related to patent foramen ovale, atrial septal defect, or coronary arteriovenous fistula (26). Hereditary thrombophilia includes factor V Leiden and protein S or C deficiency. In patients with MINOCA, the incidence of hypercoagulability is higher than in the general population (27). Several studies have demonstrated the presence of factor V Leiden in about 10% of MINOCA cases, with higher rates of genetically determined hypercoagulable state in younger patients (28–30). Acquired thrombophilia should also be considered—e.g., in antiphospholipid syndrome and myeloproliferative disorders—, and hematology profile together with transoesophageal and bubble-contrast echocardiography may be necessary to identify a specific thromboembolic cause and to inform therapy (5).
- *Anxiety and depression*: although anxiety and depression are rather frequent both in MINOCA and in MI with obstructive CAD, psychosocial disorders are more typical of MINOCA (31, 32). In a Taiwanese nationwide population-based study including more than 10,000 patients with non-obstructive CAD, over 38% had a prior anxiety disorder (33). In another study, one in five patients had a history of psychiatric disorders, and more than half reported physical and/or emotional distress within one week prior to admission, especially when diagnosed with TTS (34). Anxiety and depression may play a role in the pathogenesis of MINOCA due to their potential to trigger vasospasm and endothelial dysfunction through catecholamine surge in predisposed patients—e.g., post-menopausal women. Finally, psychiatric disorders—mainly depression—are associated with adverse clinical outcomes, MACE, and all-cause mortality in MINOCA patients as much as in patients with obstructive CAD (35).
- *Cancer*: cancer and MINOCA have a non-negligible relationship. A recent meta-analysis by Pelliccia et al. has shown that a previous diagnosis of cancer is present in about 2.5% of patients with MINOCA, which outperforms the current prevalence of malignancy in the general population (36). This observation is consistent with the results of a registry-based cohort study of patients admitted to Swedish coronary care units (37). Multiple pathophysiologic mechanisms probably play a role, including hypercoagulability, systemic inflammation, and treatment-related endothelial damage. Tumor cells can trigger coagulation through procoagulant, antifibrinolytic, and aggregating activities; release of inflammatory and angiogenic cytokines; and interaction with vascular and blood cells through adhesion molecules. Thrombophilia may also facilitate systemic embolism in atrial fibrillation (38). Enhanced inflammation and/or catecholamine release might play a role in the pathophysiology of MINOCA—i.e., epicardial or microvascular spasm (36). Finally, MINOCA may present as a complication of either chemotherapy or radiotherapy, likely due to drug-related endothelial damage. Specifically, 5-fluorouracile may induce coronary vasospasm and endothelial dysfunction. Vascular endothelial growth factor and aromatase inhibitors may favor thromboembolism. Cisplatin may induce arterial thrombosis with subsequent myocardial or cerebral ischaemia in about 2% of patients, owing to direct toxicity on the endothelium, whereas immune checkpoint inhibitors may play a role through multiple immune-related mechanisms (38, 39). Finally, several types of cancer treatment—e.g., anthracycline-, taxane-, and platinum-based chemotherapy—, increase the risk of venous thromboembolism. Thus, anti-cancer therapy may also determine MINOCA-mimicking conditions—e.g., pulmonary embolism—, making differential diagnosis very challenging in this setting.
- *Inflammatory and autoimmune diseases*: there is ample evidence that patients with chronic inflammatory and autoimmune disorders—e.g., rheumatoid arthritis, systemic lupus erythematosus, systemic sclerosis, ankylosing spondylitis, inflammatory bowel diseases, psoriasis, and periodontitis—have an increased risk of cardiovascular diseases (40). Accordingly, a meta-analysis of 41,490 patients showed that cardiovascular risk was 48% higher in patients with rheumatoid arthritis as compared to healthy individuals (41). This is partly mediated by traditional cardiovascular risk factors, but mainly depends on systemic inflammation as a promoter of endothelial dysfunction (42). Systemic lupus erythematosus and antiphospholipid syndrome have been studied most in this specific setting, as an association between them and both obstructive CAD and MINOCA was demonstrated (43). Among possible pathophysiological mechanisms, hypercoagulability can enhance thromboembolism, but microvascular dysfunction and coronary vasospasm have been described as well (41, 44). The majority of studies point to the fundamental role of systemic endothelial dysfunction in rheumatic diseases, which seems to coincide with or precede both macrovascular and microvascular involvement. Moreover, a recent observational study reported that the number of pro-inflammatory conditions—i.e., autoimmune disorders, connective tissue diseases, active cancer, and infections—was significantly higher in the MINOCA group. Nonetheless, neither all-cause mortality nor hospitalisations for cardiovascular causes differed between the groups at follow-up (31). At present, the role of inflammatory and autoimmune diseases in the development of SCAD remains controversial.
- *Infectious diseases*: acute myocardial injury is common during systemic infections—particularly pneumonia and sepsis—, and a significant association between respiratory infections—especially influenza—and acute MI was largely demonstrated (45). Coronary instability in course of an infectious illness may result from acute inflammation, biomechanical stress, and vasoconstriction. Moreover, infections may promote platelet activation and endothelial dysfunction, increase metabolic demand, and induce hypoxaemia and hypotension (46). During the coronavirus disease 2019 (COVID-19) pandemic, new challenges arose in the diagnosis and treatment of patients with acute myocardial injury or ACSs (47). Whereas severe acute respiratory syndrome coronavirus 2 (SARS-CoV-2) infection primarily affects the lungs, a heightened risk of thromboembolism has widely been reported (48). Accordingly, MINOCA has been increasingly described in COVID-19 patients, due to various mechanisms. First, increased sympathetic activation associated with viral infection may promote ACSs in patients with pre-existing CAD (47). Similarly, cytokine storm may lead to coronary plaque instability as well as initiation and perpetuation of a pro-thrombotic milieu. Furthermore, direct myocardial injury due to viral cardiomyocyte infection might play a role. Vasoconstriction and platelet activation may also occur, contributing to endothelial damage. Finally, significant hypoxia in course of viral pneumonia may not only trigger coronary plaque rupture and thrombosis, but also cause type 2 MI because of reduced oxygen supply. This deleterious pathogenic ensemble seems to be responsible for ACSs in COVID-19 (47). In addition, myocarditis, TTS, and acute heart failure are important aetiologies of myocardial injury in COVID-19, albeit non-cardiac conditions should also be considered. Sepsis—primarily originating from the lower respiratory tract—may also determine myocardial injury or type 2 MI (49). Overall, in SARS-CoV-2 infection, patients with myocardial injury have a worse prognosis and are more likely to be admitted to an intensive care unit (50). Another peculiar cause of MINOCA is coronary embolisation of an endocardial vegetation fragment. Despite its rarity—observational studies have shown that the incidence of coronary embolism during infective endocarditis approximates 0.5%—, it constitutes a potentially lethal complication (51). Oftentimes, a septic embolus is not visible on coronary angiography owing to its microvascular localisation, but should be suspected in this specific clinical setting, especially in case of multiple embolic events.
- *Pregnancy and hormonal therapy:* physiological changes associated with pregnancy and labor can facilitate SCAD, particularly in the peripartum period and in the presence of fibromuscular dysplasia or other predisposing conditions—e.g., twin gestation and pre-existing or gestational arterial hypertension—that further increase endothelial haemodynamic shear stress (52–54). Moreover, high plasma levels of both estrogen and progesterone associated not only with pregnancy, but also with hormonal contraception or hormone replacement therapy, can determine degeneration of the arterial intima-media layer in predisposed individuals, mainly through a combination of decreased release of extracellular matrix and production of matrix metalloproteinases (14, 55, 56). MINOCA due to coronary thrombosis without CAD is a rather frequent cause of pregnancy-related MI, mainly depending on the hypercoagulable state related to gestation (57, 58). Moreover, pre-eclampsia heightens the risk of coronary spasm, since it provokes endothelial dysfunction due to an imbalance in the release of endothelin and thromboxane (59). Pregnancy-related coronary spasm may also result from enhanced vascular reactivity to some hormones, such as angiotensin II and noradrenaline (60).

## Conclusions

The definition of MINOCA groups several conditions that share the absence of obstructive coronary arteries. As such, under the MINOCA umbrella, each pathologic process shows its own peculiarities. Unfortunately, all the available data are currently retrospective and have limited quality, often showing evident contradictions. There is utter need for further clarification of possible mechanisms and prognostic implications in specific subsets of patients. Accordingly, as MINOCA still remains somewhat indefinite and nebulous, the identification of the underlying disease and associated comorbidities is paramount for an in-depth understanding of pathophysiology, prevention, and specific treatment targets.

## Author Contributions

AM, VG, and IP contributed to conception and design of the study. AM wrote the first draft of the manuscript. AM, AT, EP, and VG wrote sections of the manuscript. All authors contributed to manuscript revision, read, and approved the submitted version.

## Conflict of Interest

The authors declare that the research was conducted in the absence of any commercial or financial relationships that could be construed as a potential conflict of interest.

## Publisher's Note

All claims expressed in this article are solely those of the authors and do not necessarily represent those of their affiliated organizations, or those of the publisher, the editors and the reviewers. Any product that may be evaluated in this article, or claim that may be made by its manufacturer, is not guaranteed or endorsed by the publisher.
